# Clinical relevance of the combined analysis of circulating tumor cells and anti-tumor T-cell immunity in metastatic breast cancer patients

**DOI:** 10.3389/fonc.2022.983887

**Published:** 2022-08-23

**Authors:** Elena Muraro, Fabio Del Ben, Matteo Turetta, Daniela Cesselli, Michela Bulfoni, Rita Zamarchi, Elisabetta Rossi, Simon Spazzapan, Riccardo Dolcetti, Agostino Steffan, Giulia Brisotto

**Affiliations:** ^1^ Immunopathology and Cancer Biomarkers Units, Department of Translational Research, Centro di Riferimento Oncologico di Aviano (CRO), Istituto di Ricovero e Cura a Carattere Scientifico, Aviano, Italy; ^2^ Department of Medicine, University of Udine, Udine, Italy; ^3^ Institute of Pathology, University Hospital of Udine (Azienda sanitaria universitaria Friuli Centrale, ASUFC), Udine, Italy; ^4^ Department of Surgery, Oncology & Gastroenterology, University of Padova, Padua, Italy; ^5^ Veneto Institute of Oncology IOV - Istituto di Ricovero e Cura a Carattere Scientifico, Padua, Italy; ^6^ Medical Oncology and Cancer Prevention Unit, Centro di Riferimento Oncologico di Aviano (CRO), Istituto di Ricovero e Cura a Carattere Scientifico, Aviano, Italy; ^7^ Peter MacCallum Cancer Centre, Melbourne, VIC, Australia; ^8^ Sir Peter MacCallum Department of Oncology, The University of Melbourne, Melbourne, VIC, Australia; ^9^ Department of Microbiology and Immunology, The University of Melbourne, Melbourne, VIC, Australia; ^10^ The University of Queensland Diamantina Institute, Brisbane, QLD, Australia

**Keywords:** metastatic breast cancer (mbc), liquid biopsy, circulating tumor cells (CTCs), anti-tumor T-cells, T-cell receptor (TCR)

## Abstract

**Background:**

Metastatic breast cancer (mBC) is a heterogeneous disease with varying responses to treatments and clinical outcomes, still requiring the identification of reliable predictive biomarkers. In this context, liquid biopsy has emerged as a powerful tool to assess in real-time the evolving landscape of cancer, which is both orchestrated by the metastatic process and immune-surveillance mechanisms. Thus, we investigated circulating tumor cells (CTCs) coupled with peripheral T-cell immunity to uncover their potential clinical relevance in mBC.

**Methods:**

A cohort of 20 mBC patients was evaluated, before and one month after starting therapy, through the following liquid biopsy approaches: CTCs enumerated by a metabolism-based assay, T-cell responses against tumor-associated antigens (TAA) characterized by interferon-γ enzyme-linked immunosorbent spot (ELISpot), and the T-cell receptor (TCR) repertoire investigated by a targeted next-generation sequencing technique. TCR repertoire features were characterized by the Morisita’s overlap and the Productive Simpson Clonality indexes, and the TCR richness. Differences between groups were calculated by Fisher’s, Mann-Whitney or Kruskal-Wallis test, as appropriate. Prognostic data analysis was estimated by Kaplan-Meier method.

**Results:**

Stratifying patients for their prognostic level of 6 CTCs before therapy, TAA specific T-cell responses were detected only in patients with a low CTC level. By analyzing the TCR repertoire, the highest TCR clonality was observed in the case of CTCs under the cut-off and a positive ELISpot response (p=0.03). Whereas, at follow-up, patients showing a good clinical response coupled with a low number of CTCs were characterized by the most elevated TCR clonality (p<0.05). The detection of CTCs≥6 in at least one time-point was associated with a lower TCR clonality (p=0.02). Intriguingly, by combining overall survival analysis with TCR repertoire, we highlighted a potential prognostic role of the TCR clonality measured at follow-up (p=0.03).

**Conclusion:**

These data, whether validated in a larger cohort of patients, suggest that the combined analysis of CTCs and circulating anti-tumor T-cell immunity could represent a valuable immune-oncological biomarker for the liquid biopsy field. The clinical application of this promising tool could improve the management of mBC patients, especially in the setting of immunotherapy, a rising approach for BC treatment requiring reliable predictive biomarkers.

## Introduction

Breast cancer (BC) is the most commonly diagnosed cancer among females worldwide, accounting for 24.5% of all new cancer cases (1). Over the past two decades, BC mortality rates have steadily declined, along with overall improved survival (2). This is mainly due to screening programs for early cancer detection, adjuvant therapies to reduce distant recurrence risk, and more effective therapeutic options for the metastatic stage (3). However, patients with metastatic BC (mBC) are typically incurable, and metastases are the leading cause of BC death. MBC is a heterogeneous disease with varying responses to treatments and clinical outcomes (4). Thus, the identification of effective prognostic factors, the optimal sequence of treatments, and an understanding of immune surveillance remains a challenging clinical need (5).

Liquid biopsy, defined as the sampling and analysis of tumor-derived analytes [i.e.: circulating tumor cells (CTCs), circulating tumor DNA (ctDNA) or exosomes] from blood, has recently emerged as a powerful tool to assess in real-time the evolving landscape of cancer, identify prognostic and predictive biomarkers and detect resistance to therapies in several cancers, including BC (6). Additionally, the same liquid blood sample represents a useful resource for simultaneously profiling also tumor-associated components, such as circulating immune cells, which may give clues at the systemic level about the dynamic and complex host-tumor interaction (7, 8). It is non-invasive, easily repeatable, and cost-effective.

CTCs, those cancer cells that detach from a solid tumor lesion and enter the bloodstream, are widely recognized as precursors of metastasis (9). At present, the CellSearch system (Menarini Silicon Biosystems, Bologna, Italy) is the only FDA-approved technology for CTC enumeration as an aid for mBC monitoring. It defines CTCs as Epithelial cell adhesion molecule (EpCAM)- and Cytokeratine-positive, and CD45-negative cells (10). With this technology, several works have demonstrated that the level of CTCs is a valuable prognostic factor of worse outcomes and CTCs dynamics can predict treatment response (10–12). Further, recent evidence suggests the feasibility for a CTC-driven treatment choice (12). To note, CellSearch is limited to EpCAM-positive cells, and several studies showed how downregulation of EpCAM and epithelial-mesenchymal transition occur often in cancer and might reduce detection rate (11). Indeed, several groups have reported about the presence of non-epithelial CTC and their correlation with worse prognosis and resistance to therapies (9). For this major drawback, new technologies for CTC enrichment and enumeration are under development, as for example size-based methods, which however are limited by a low specificity (11). Besides their clinical significance, CTCs isolation and characterization offer the opportunity for probing the biological evolution of cancer towards the metastatic stage (7). In this regard, the Parsortix system (13) recently reached the FDA-approval for the capture and harvest of CTCs from mBC patients.

Once in the bloodstream, only a small subgroup of CTCs is able to survive the harsh conditions of the blood microenvironment and successfully colonize a distant site (14, 15). Besides factors such as shear stress and anoikis, several studies suggest that CTCs survival might be hindered by mechanisms of immune-mediated clearance (8, 16). Research interest in understanding the interplay between CTCs and circulating immune cells is growing as it holds great promise to understand the process of metastasis, predict patients’ outcome and pave the way for new treatment strategies (16–18).

T-cells are widely recognized as critical players in anti-tumor immunity. Indeed, the presence of Tumor-Infiltrating Lymphocytes (TILs) has been correlated with good prognosis and treatment response in BC (19–21). Further, assessing peripheral lymphocyte count and function has shown its potential as a predictive and prognostic tool (22–25). In-depth analyses of circulating T-cells could provide useful information on the host’s immune status, and, indirectly, also on their ability to combat the tumor.

T-cell activation is initiated by the recognition of peptide epitopes presented on the major histocompatibility complex (MHC) molecules through the T-cell receptor (TCR) (26). T-cell antigen specificity mainly depends on the CDR3 region variability, that is the product of the so-called V(D)J somatic recombination, whereas a successful T-cell response relies on the existing TCR repertoire, defined as the number of T-cells clones with a distinct TCR (26). Therefore, the TCR repertoire plays a major role in the definition of the individual’s immune status. Recent studies in various cancer types suggested that the assessment of TCR diversity, clonality and dynamic changes on circulating T-cells during therapy is a valuable tool to estimate the anti-tumor activity, define the interaction between host and tumor and predict therapy response (27, 28).

Regarding the relationship between CTCs and the peripheral adaptive immunity in BC, data are limited, but available evidence suggests an impaired immunity response in presence of CTCs (29–31).

On these grounds, the present study aimed at investigating the association of the anti-tumor activity of circulating T-cells and the level of CTCs before and after treatment in a cohort of mBC patients and to evaluate their potential predictive and prognostic role. For this cohort, we previously reported that an increased level of dysmetabolic CTCs, as detected by a new CTC assay developed by our group, was associated with a worst prognosis and that tracking CTC dynamic over time improved patient stratification (32). With the final goal to further improve the predictive and prognostic definition of mBC patients, here we investigated the clinical relevance of integrating CTC data with T-cell responses against BC associated antigens and the variations of TCR repertoire assessed in the peripheral blood.

## Materials and methods

### Patient assessments and therapy

The study was conducted at the IRCCS-CRO Aviano-National Cancer Institute and approved by the Institutional Review Board with number IRB-12-2014 (32). Informed and written consent was obtained from all patients and healthy donors before their enrolment, and their clinico-pathological information was recorded. Twenty patients with the following inclusion criteria were consecutively enrolled: progressive and measurable stage IV mBC; beginning a new systemic therapy; no limits to round and type of previous therapies (hormone therapy, chemotherapy, targeted therapy). Primary tumor receptor status for Estrogen Receptor (ER) and/or Progesterone Receptor (PgR) was detected by Immunohistochemistry (IHC) and Human Epidermal Growth Factor Receptor 2 (HER2) expression was evaluated by IHC or Fluorescent *In Situ* Hybridization (FISH). All patients had an Eastern Cooperative Oncology Group performance status (ECOG PS) score ≤ 1. Before starting a new therapy, patients underwent a baseline (T0) blood drawn for CTC evaluation and routine clinical tests. Another blood sample was collected 3–4 weeks after the beginning of the therapy (follow-up, T1). Clinical re-evaluation of the disease status was conducted depending on the type and schedule of the therapy; Standard Response Evaluation Criteria in Solid Tumors (RECIST) criteria were used to determine patients’ responses to treatment. This study did not interfere with routine imaging schedule, nor imposed a uniform imaging schedule to all patients. The follow-up imaging schedule was decided by the oncologists on an individual basis. For most patients entered in the study, CT of the chest and abdomen, or PET, approximately every 3–6 months, were performed. In selected cases e.g., in the case of liver metastases as the unique site of disease, the disease parameter was simplified by using a liver ultrasound. Overall Survival (OS) was defined as the time intercourse from baseline to death or 24 months of follow-up.

### CTC detection

A detailed description of the procedure employed for the enrichment and identification of CTCs with a metabolism-based assay (MBA) has been published previously (32). Briefly, peripheral blood samples (2.5 ml) were drawn into K2-EDTA Vacutainer tubes (Becton Dickinson). After red blood cell lysis with the BD Pharm Lyse lysing solution (Becton-Dickinson, Franklin Lakes, NJ, USA), samples were immune-depleted of CD45-positive WBCs and residual red blood cells using CD45 and Glycophorin-A microbeads (Miltenyi Biotec, Bergisch GladBack, Germany), respectively, and LD separation columns in a MACS MIDI separator (Miltenyi Biotec), according to the manufacturer’s instructions. The CD45-negative fraction was stained with anti-CD45 (BD Horizon Brilliant™ Violet 480, dilution 1:100) and anti-EpCAM (BD Horizon Brilliant™ Violet 421, dilution 1:100) and resuspended in 50 μL of an unbuffered Joklik’s modified EMEM culture medium (Sigma) containing 2 mM EDTA, 0.1% BSA, 15% Optiprep and 4mM of the fluorescent pH indicator SNARF-5F (Thermo Fisher Scientific). Then, cells were single-cell encapsulated in monodisperse droplets using a droplet microfluidic platform and incubated at 37°C for 30 min. During in-drop incubation each cell extrudes a certain quantity of H+ depending on its metabolism, altering the pH of the droplet, which is then assessed by optical measurement. Beside pH determination, the system allows to determine EpCAM and CD45 expression, and to capture images of the abnormal cells. Positive events were defined as CD45-negative cells able to acidify their extracellular environment to a pH lower than 6.4 (MBA-CTC). The number of CTCs was then proportionally adjusted to 7.5 mL as the commonly used blood sample volume for CTC analysis; we previously have shown that this normalization does not affect patient classification at the defined cut-off (32). All evaluations were performed without knowledge of the clinical status of the patients.

### Sample collection and storage

Blood samples were collected from 20 mBC patients included in the study before and 3-4 weeks after starting the treatment and from 5 healthy women as controls. Peripheral blood mononuclear cells (PBMCs) were freshly isolated (within 5 hours after blood drawing) from blood samples collected in EDTA before therapy by Ficoll-Hypaque gradient (Lymphoprep, Fresenius Kabi Norge Halden) using standard gradient separation. Cells were washed in PBS (Biomerieux), counted using an automated cell counter (ADAM-MC™, DigitalBio, NanoEnTek Inc.) and viably frozen [90% heat-inactivated Fetal Bovine Serum (FBS; Euroclone) and 10% DMSO] at -80°C for 24 h and then in liquid nitrogen until use. After thawing in IMDM (Lonza) containing 2 mM L-glutamine, 100 μg/ml streptomycin and 100 IU/ml penicillin (Sigma-Aldrich), supplemented with 2% human serum (Sigma-Aldrich) and with 3 μg/ml Deoxyribonuclease (Sigma-Aldrich), cells were washed in PBS (Biomerieux) and counted again to check viability (>80%). Five hundred µl buffy coat samples were obtained from 7.5 ml EDTA-blood samples collected after therapy and centrifuged at 3600 rpm for 10 min, then maintained at -80°C until use.

### Peptides mixes and IFN-γ ELISpot assay

Tumor associated antigens (TAA)-specific T-cell responses were investigated by using an interferon (IFN)-γ enzyme-linked immunosorbent spot (ELISpot) commercial assay [“Human IFN-γ Single Color ELISPOT”, ImmunoSpot^®^, Cellular Technology Limited (CTL), OH, USA], according to the manufacturer’s instructions and employing commercial peptides mixes as stimulators. We selected Survivin, Mammaglobin A, and HER2 as breast cancer associated antigens inducing a T-cell response based on previous evidence (24). The Survivin-derived “ProMix™ Survivin Peptide Pool” and the CMV/EBV/FLU CEF-derived “ProMix™ CEF peptide pool”, purchased from Thinkpeptides ProImmune (Oxford, UK), were diluted in DMSO 10% in PBS at a final concentration of 10 μg/ml, and stored frozen. The Mammaglobin-A-derived “PepMix™ Human (Mammaglobin A)”, and the HER2-derived “PepMix™ Human (ErbB2_ECD)”, purchased from JPT Peptide Technology (Berlin, Germany), were diluted in DMSO at a final concentration of 0.5 mg/ml and stored frozen.

The IFN-γ ELISpot assay was carried out after overnight pre-coating of plates with the Human IFN-γ Capture Solution at 4°C. The next day, PBMCs were thawed and washed once in serum free RPMI-1640 (Gibco, Life Technologies, Paisley, UK), counted and resuspended in CTL-test Medium (CTL, Immunospot, Bonn, Germany) at a final concentration of 3x10^6/mL cells. The PepMix antigen solutions were seeded onto ELISpot capture plates in triplicates and incubated 20 minutes at 37°C with 5% CO_2_. Patient’s PBMCs were then plated (300,000 cells/well) in triplicates and incubated for 24 h at 37°C with 5% CO_2_. Medium and PBMCs supplemented with DMSO were used as negative controls, while the CEF peptide pool and unspecific stimuli with 0.5mg/mL α-CD3 and α-CD28 antibodies were used as positive controls. The next day, spots were detected with anti-human IFN-γ (biotin) streptavidin alkaline phosphatase, and Blue Developer Solution. All plates were then evaluated by a computer-assisted ELISpot reader (CTL Immunospot^®^ plate scanning and analysis service, CTL-Europe GmbH, Bonn, Germany). The number of spots in negative control wells was subtracted from the number of spots in stimulated wells. Responses were considered positive if a minimum of six IFN-γ producing cells were calculated for each well.

### DNA extraction and molecular analyses

For TCR sequencing, genomic DNA was extracted from frozen PBMCs (ranging from 1.6 to 7.3x10e6 total number of cells/sample) or buffy coat samples (150 µL) by using QIAamp DNA Blood kit (Qiagen) according to the manufacturer’s protocol. The concentration and quality of isolated DNA were assessed using a NanoDrop 2000c spectrophotometer (Thermo Fisher Scientific, USA). The extracted DNA was used for the deep resolution sequencing of the CDR3 regions of human TCR-β chains with the ImmunoSEQ Assay (Adaptive Biotechnologies, Seattle, Washington, USA), following manufacturer’s instructions. Briefly, the CDR3 region of TCRs is amplified using a bias-controlled, multiplex PCR method (33, 34). Sequencing was performed on an Illumina NextSeq 550 system with the NextSeq 500 Mid Output (150 cycles) reagent kit (Illumina, San Diego, CA) and the run parameters as recommended by ImmunoSEQ’s manufacturer. Raw data were uploaded to the ImmunoSEQ platform for initial bioinformatics analysis. Processed data were accessed for further analysis throughout the ImmunoSEQ Analyzer 3.0 software from Adaptive Biotechnologies.

### Statistical analysis

Data were expressed as single values and box plots, where the horizontal line represents the median value, the box the interquartile range, and the whiskers the lower and the higher value included in the following interval: 1st quartile - 1.5x(3rd-1st quartile) and 3rd quartile + 1.5x(3rd -1st quartile). Raw data can be provided per request. The Fisher’s exact test was used to evaluate association between clinical-pathological parameters and CTCs, and a positive response to TAA and CTC numbers below or above the threshold value. The Morisita’s overlap index was calculated to determine similarities between samples, ranging from 0, as minimal, and 1, as maximal similarity. The TCR clonality was evaluated through the Productive Simpson Clonality, which in turn is calculated as the square root of Simpson’s diversity index for all productive rearrangements. Values range between 0, representing a polyclonal population, to 1, a monoclonal sample. The TCR richness was instead expressed as total productive templates, meaning those rearrangements that can produce a functional protein receptor (in frame and not containing a stop codon). The presence of expanded or contracted clones between paired samples was verified through the Differential abundance tool of the ImmunoSEQ Analyzer 3.0 software, and then compared through the Student’s t test for two tailed distributions and paired data. The correlation between age and TCR clonality was measured using the Spearman’s correlation coefficient. Differences among groups obtained by stratifying patients based on CTC below or above the cut-off value, positive/negative immune response to TAA, and/or clinical response to therapy were evaluated through the Grouped Comparison tool of the ImmunoSEQ Analyzer 3.0 software. Briefly, the software analysis performed a Mann-Whitney U test to compare two groups, or a Kruskal-Wallis test to compare more than two groups, followed by the Dunn’s test for the pairwise comparison between samples. For survival analysis the global cohort of patients was divided into two groups based on the TCR clonality median value, to identify “high” and “low” subgroups. OS was estimated by Kaplan-Meier plots, starting from the baseline date to the event or the last follow-up available. The Log-rank test was used to compare the survival curves. Differences were considered statistically significant when P ≤ 0.05.

## Results

### Patients’ and tumor characteristics

We previously reported CTC analysis on a cohort of 31 mBC patients (32), 20 of whom were selected based on the availability of peripheral lymphocytes samples for further characterization of their immune status in the present study. Patients were mainly women (19 out of 20, 95%), with a median age of 56 years (40–75). **Table 1** and **Supplemental Table 1** shows the main clinical-pathological parameters of the global case study and the distribution of CTCs based on these parameters. The majority of patients carried luminal BC subtype (13 out of 20, 65%), a Triple Negative BC (TNBC) subtype was reported in 6 out of 20 of patients (30%), while only 1 (5%) patient had a HER2+ tumor. Almost half (9 out of 20, 45%) of patients showed more than 2 metastatic sites, the preferential sites of metastasis were bone and nodes, followed by liver and lung, only one patient had a brain metastasis. Patients underwent different chemotherapeutic regimens, most of which included chemotherapy alone (17 out 20, 85%), and half of the cases had already been treated with more than 3 therapy cycles. A complete or partial clinical response to therapy was reported in 10 out of 20 patients (50%).

**Table 1:** Patients and tumor characteristics.

Clinical-pathological features	Data description	CTC<6	CTC ≥6
**Age**	*Median (range)* 56 (40-75)	*Median (range)* 56 (43-74)	*Median (range)* 63 (40-75)
**Sex**	*n (%)*	*n (%)*	*n (%)*
Female	19 (95)	12 (60)	7 (35)
Male	1 (5)	0 (0)	1 (5)
**Tumor molecular subtype**	*n (%)*	*n (%)*	*n (%)*
Luminal	13 (65)	9 (45)	4 (20)
HER2+	1 (5)	1 (5)	0 (0)
TNBC	6 (30)	2 (10)	4 (20)
**Number of metastatic sites**	*n (%)*	*n (%)*	*n (%)*
1	4 (20)	3 (15)	1 (5)
2	7 (35)	5 (25)	2 (10)
>2	9 (45)	4 (20)	5 (25)
**Metastatic site**	*n (%)*	*n (%)*	*n (%)*
bone	16 (80)	10 (50)	6 (30)
nodes	14 (70)	7 (35)	7 (35)
liver	9 (45)	5 (25)	4 (20)
lung	8 (40)	4 (20)	4 (20)
brain	1 (5)	0 (0)	1 (5)
**Type of therapy**	*n (%)*	*n (%)*	*n (%)*
Chemotherapy alone	17 (85)	10 (50)	7 (35)
Chemotherapy and targeted therapy	2 (10)	2 (10)	0 (0)
Placebo	1 (5)	0 (0)	1 (5)
**Nr of previous treatments**	*n (%)*	*n (%)*	*n (%)*
1	5 (25)	7 (35)	2 (10)
2	3 (15)	0 (0)	1 (5)
≥ 3	11 (55)	1 (5)	4 (20)
n.a.	1 (5)	0 (0)	1 (5)
**Type of previous treatments**	*n (%)*	*n (%)*	*n (%)*
Chemotherapy	2 (10)	1 (5)	1 (5)
Chemotherapy and hormone therapy	8 (40)	5 (25)	3 (15)
Chemotherapy and targeted therapy	3 (15)	1 (5)	2 (10)
Chemotherapy, hormone therapy and targeted therapy	4 (20)	4 (20)	0 (0)
Hormone therapy	1 (5)	1 (5)	0 (0)
Chemotherapy, hormone therapy and immunosuppressant	1 (5)	0 (0)	1 (5)
Chemotherapy, hormone therapy, targeted therapy and immunosuppressant	1 (5)	0 (0)	1 (5)
**Previous radiotherapy**	*n (%)*	*n (%)*	*n (%)*
Yes	10 (50)	5 (25)	5 (25)
No	10 (50)	7 (35)	3 (15)
**Therapy Response**	*n (%)*	*n (%)*	*n (%)*
CR	1 (5)	1 (5)	0 (0)
PR	9 (45)	8 (40)	1 (5)
SD	1 (5)	1 (5)	0 (0)
PD/none	9 (45)	2 (10)	7 (35)

CTC, Circulating Tumor Cells; HER2, Human Epidermal Growth Factor Receptor 2; TNBC, Triple Negative Breast Cancer; CR, Complete Response; PR, Partial Response; SD, Stable Disease; PD, progressive disease.

The presence of CTCs was evaluated by using a metabolism-based assay (MBA). At baseline, patients presenting at least one CTCs were 8 (40%) out of 20, all showing a level of CTCs above the cut-off of 6 cells. Overall, the median CTC count was 0 [interquartile range (IQR) 0-16, min 0-max 5319] (**Table 2** and **Supplementary Table 2**). At follow-up, among the 11 (61%) out of 18 mBC patients with detectable CTCs, 4 (22%) had an MBA-CTC count ≥ 6 CTCs. The median CTC count was 4 (IQR 0-5, min 0-max 280) (**Table 2** and **Supplementary Table 2**). CTC levels were correlated with the number of previous treatments and with therapy response (**Supplementary Table 1**) (32).

**Table 2:** Prevalence of CTCs before and after treatment in mBC patients.

Cohort	N		Median (IQR)	Range (min-max)	% patients with CTCs ≥6
**mBC at T0**	20		0 (0-16)	0-5319	40%
**mBC at T1**	18		4 (0-5)	0-280	22%

T0, baseline; T1, follow-up; mBC, metastatic breast cancer; CTCs, circulating tumor cells; IQR, interquartile range.

### Monitoring of T-cell responses against Tumor Associated Antigens before treatment

The presence of spontaneous T-cell responses to the BC-associated antigens Survivin, Mammaglobin-A, and HER2, was investigated by IFN-γ ELISpot assay after stimulation of patients’ PBMCs through peptide mixes derived from the single tumor-associated antigens (TAAs). The analysis was carried out in blood samples of 20 patients collected before treatment (**Supplementary Table 2**) and in 5 healthy women as controls.

**Figure 1:**
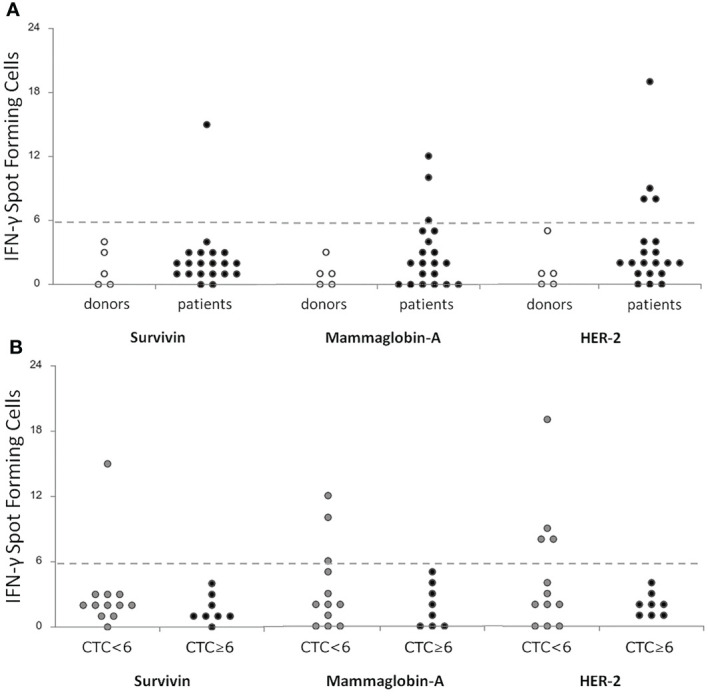
Correlation of T-cell specific responses against breast-tumor associated antigens (Survivin, Mammoglobin-A and HER2) between a cohort of donors (n = 5) and metastatic breast cancer patients (n = 20) before treatment **(A)** and between patients stratified according to the level of CTCs <6 (n = 12) or ≥6 (n = 8) **(B)**. T-cell responses were considered positive if at least 6 IFN-γ spot forming cells were detectable. CTC, Circulating Tumor Cells; IFN-γ, Interferon-gamma.

A positive T-cell response was observed in 1/20 (5%) patients for Survivin, in 3/20 (15%) patients for Mammaglobin-A, and in 4/20 (20%) patients for HER2, while no healthy donors showed TAA-specific immunity (**Figure 1A**). T-cell responses against the positive control CEF were registered in all but 3 patients and in all but 1 healthy donor (not shown), while the unspecific stimulation with α-CD3 and α-CD28 antibodies induced a positive signal in all cases (not shown).

Globally, 5/20 (25%) patients showed a positive response to at least one TAA (**Supplementary Table 2**). Interestingly, when correlating these data with the CTC analysis, we noticed that a positive response to TAA was highlighted in the 42% of patients showing CTC below the cut-off (5 out of 12) (32), while in cases with CTC above the cut-off no specific T-cell responses against TAA were detectable (marginally significant, Fisher’s exact test, p=0.05, **Table 3**, **Figure 1B**). No correlations were observed between TAA-specific T cell responses and clinical variables (data not shown).

**Table 3:** TAA-specific T-cell responses based on CTC analysis.

		TAA-specific immunity		
		Neg	Pos	p-value
**CTC**	<6	7 (58%)	5 (42%)	0.05
	≥6	8 (100%)	0 (0%)	

CTC, Circulating Tumor Cells; TAA, Tumor-Associated Antigens; neg, negative; pos, positive.

### Characterization of the TCR repertoire at baseline and follow-up

Twelve out of 20 patients (60%) were further characterized by TCRB NGS analysis, based on biological sample availability. For all but one patient, we conducted the analysis on samples collected before (baseline PBMCs) therapy, and in all of them 3–4 weeks after (follow-up buffy coat) starting a new systemic therapy. Globally, 4330618 productive templates were obtained from a total of 23 blood samples. The average count of total rearrangements/sample was 139613 ± 61221 (range 42496-278907), while unique clonotype per sample was 113044 ± 50408 (range 33138-222605).

We further proceeded through the characterization of the TCR repertoire evaluating the following parameters: the number of TCR shared between samples, i.e., the same immune specificities (Morisita index); the frequency variation of each TCR sequence, i.e., the distribution of specific T-cell clones (Simpson clonality); the number of TCR sequences able to code for a functional TCR, i.e., all the T-cell specificities within a sample (TCR richness).

Paired samples obtained from the same patient at different time points showed an average Morisita similarity index of 0.90 ± 0.10 (Morisita index range 0-1, with 1 max similarity; **Supplementary Figure 1**). Conversely, comparing samples from different patients we detected Morisita similarity indexes < 0.00012, thus suggesting a high degree of diversity. The TCR clonality was evaluated through the Simpson Clonality index, which accounted for a median value of 0.024 (min 0.009-max 0.205) and 0.037 (min 0.008-max 0.219) at baseline and follow-up, respectively (**Supplementary Table 2**).

No correlation was observed between age and TCR clonality neither at baseline nor at follow-up (not shown). The tumor subtype seemed to influence the TCR richness (positively correlated with the number of total productive templates), with luminal tumors showing an improved number of total productive templates compared to HER2+ or TNBC malignancies (**Supplementary Figure 2**). Baseline TCR clonality was not dependent on the number or the localization of metastatic sites, nor the number of previous treatments (not shown).

Comparing the differential abundance of T-cell clones between paired samples, we noticed a significantly increased number of expanded clones at follow-up (18 ± 15 at baseline; 66 ± 61 at follow-up; p=0.04; **Supplementary Figure 3**), thus suggesting a potential role of therapy in the proliferation of selected T-cell clonal populations. However, the type of therapy did not influence the TCR clonality measured at follow up (not shown).

**Figure 2:**
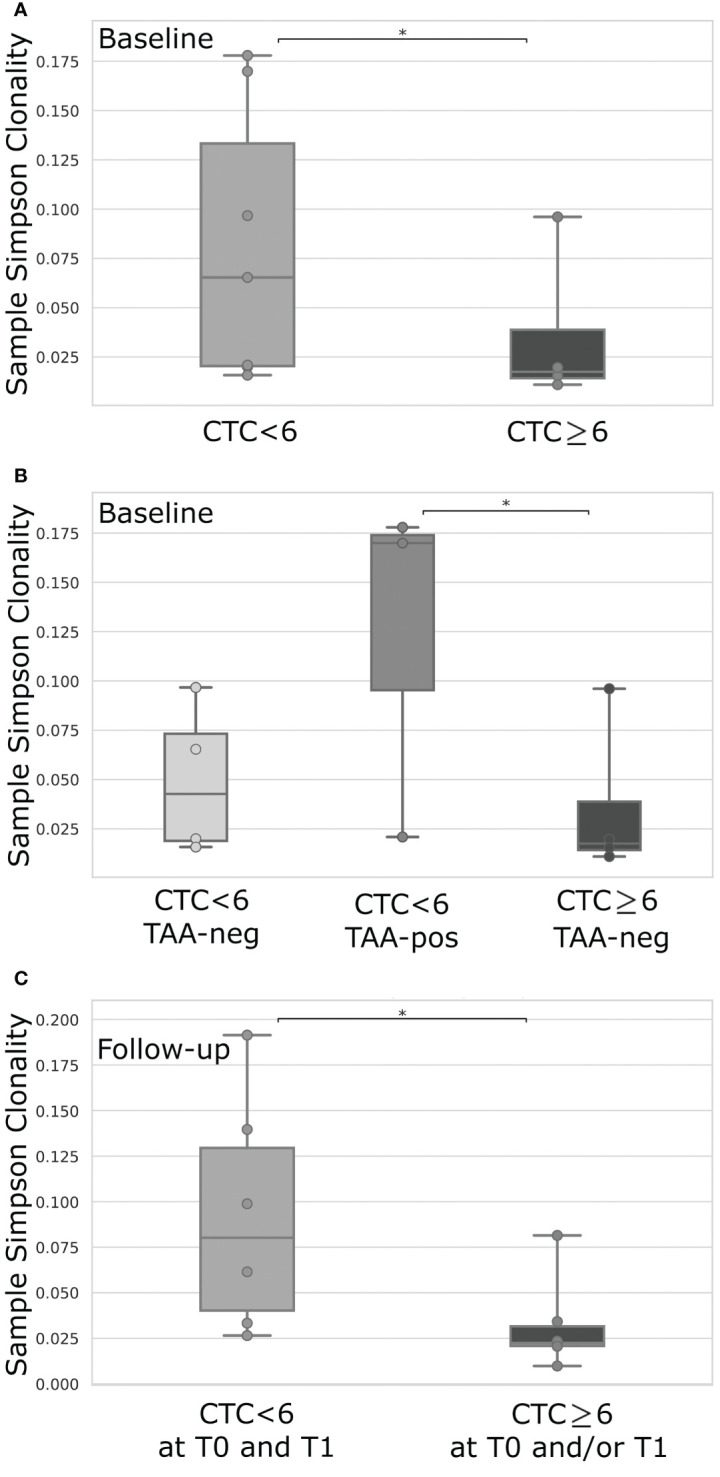
TCR clonality comparison of mBC patients dichotomized for their level of CTCs <6 (n = 6) or ≥6 (n = 4) **(A)**, or according to level of CTCs <6 or ≥6 combined with the positive (pos) or negative (neg) T-cell response against breast cancer TAA **(B)**, or based on the level of CTCs <6 at both time-points (T0 and T1) or ≥6 in at least one time-point (T0 and/or T1) **(C)**. TAA-response was classified as positive if 6 or more IFN-γ spot forming cells were detectable for at least one of the investigated TAAs (Survivin, Mammoglobin-A, HER2). For each box plot, points represent the value of a single sample. CTC, Circulating Tumor Cells; IFN-γ, Interferon-gamma; TAA, Tumor-Associated Antigens; *p-value < 0.05.

Stratifying patients for their level of CTCs before therapy, we interestingly noticed that cases with CTCs under the cut-off value presented a higher TCR clonality compared to patients characterized by a level of CTCs above the threshold (p=0.04; **Figure 2A**). This difference was not evident at follow-up (not shown). Intriguingly, in case of both CTC under the threshold and positive response to TAA, the TCR clonality appeared even higher to those of patients presenting CTC above the cut-off and no anti-TAA immune response (p=0.03; **Figure 2B**). Further, classifying patients in two groups, as having (or not having) at least one time-point with CTCs above the threshold, we noticed a higher TCR clonality at follow-up in the absence of CTCs at both time points compared to cases showing at least one evidence of CTCs (p=0.02; **Figure 2C**).

### Association of the TCR repertoire with patients’ clinical data

**Figure 3:**
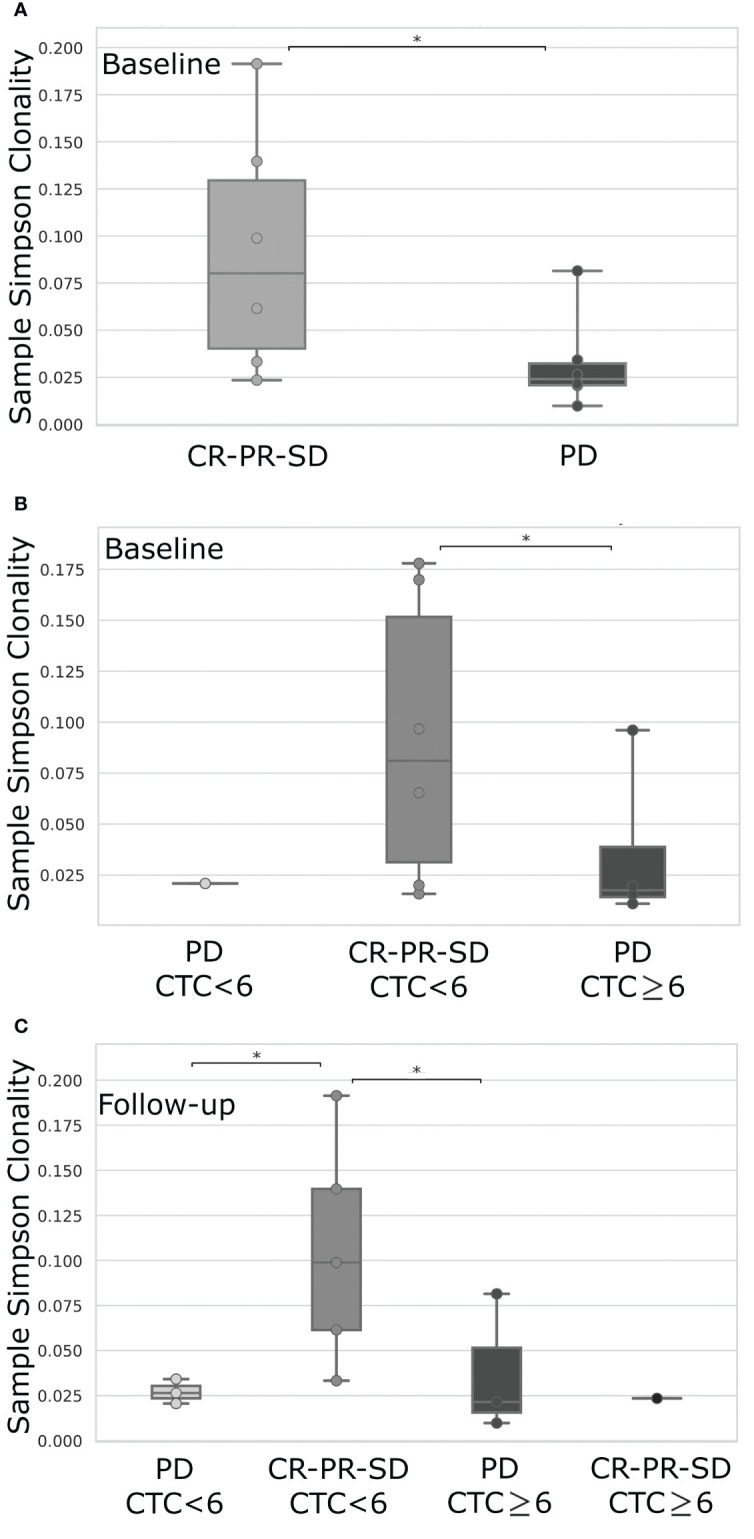
TCR clonality comparison between mBC patients stratified according to their favorable (CR, Complete Response; PR, Partial Response; SD, Stable Disease) or unfavorable response (PD, Progressive Disease), evaluated at the first follow-up imaging **(A)**, or first follow-up imaging data combined with the level of CTCs <6 or ≥6 as detected at baseline **(B)** or follow-up **(C)** time-point. CR, Complete Response; PR, Partial Response; SD, Stable Disease; PD, Progressive Disease; CTC, Circulating Tumor Cells. *p-value< 0.05.

We finally correlated the TCR repertoire and the CTC analysis with patients’ clinical outcome. Interestingly, grouping patients for their clinical response to therapy we observed a higher TCR clonality, measured at follow-up, in case of complete or partial response, or stable disease compared to progression disease (p=0.03; **Figure 3A**).

Further stratifying patients by CTC analysis together with therapy response, we noticed an improved TCR clonality at baseline in the presence of CTC under the cut-off value and favorable clinical response, compared to progressive disease associated with a higher number of CTCs (p=0.04; **Figure 3B**). Intriguingly, the most striking difference was observed at follow-up where the highest TCR clonality was detected in those patients characterized by CTC below the threshold value and a good clinical response, if compared to all the other groups (p<0.05; **Figure 3C**).

**Figure 4:**
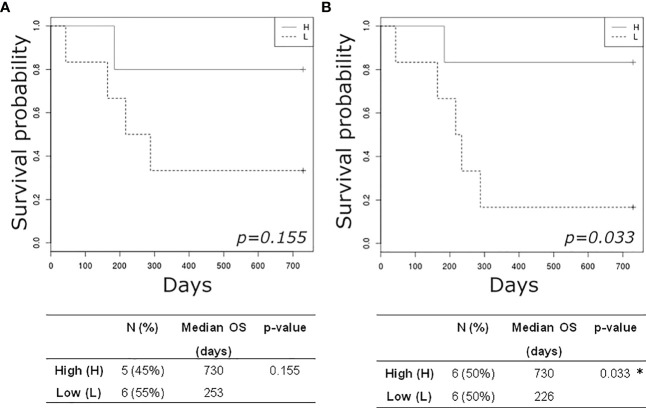
Kaplan-Meier plots estimating overall survival of mBC patients according to high **(H)** or low (L) TCR clonality index detected at baseline **(A)** and at follow-up **(B)**. *p-value< 0.05.

Finally, dichotomizing patients by the baseline median Simpson Clonality Index in High (H) versus Low (L) cases we observed an improved, but not significant, 2-year OS in the H group (**Figure 4A**). Likewise, this trend was maintained and reached significance when evaluated at follow-up (**Figure 4B**), thus suggesting the prognostic value of this analysis.

## Discussion

In the present pilot study, we performed a combined analysis of CTC and circulating T-cells in a cohort of mBC patients, with the aim to investigate the interplay between cancer and anti-tumor T-cell immunity by exploiting the peripheral blood as the most accessible source to monitor disease evolution and clinically relevant parameters. Despite the limited number of patients included, the present analysis could represent an hypothesis-generating study highlighting interesting points on the cancer-immunity liaison.

We interestingly noticed that systemic evidence of functional anti-tumor T-cell responses was detectable only in patients showing a CTC number below the prognostic cut-off value, even if the low number of patients investigated did not allow to reach conclusive results. However, these observations are consistent with the association of immune suppression with disease progression (35) and with the hijacking of the anti-tumor immunity promoting the tumor metastatic stage (36). In line with this observation, high CTCs count and lymphocytopenia were independent prognostic factors of poor prognosis in mBC patients (29). Further, inflammatory BC patients with detectable numbers of CTCs showed a lower percentage of CD3+ and CD4+ T-cells and CD8+ T-cells synthesizing TNF-α and IFN-γ compared to patients with no CTCs (30).

Consistently with our previous findings, effective TAA-specific T-cells were more frequently detected in patients compared to donors even if not all patients showed a specific immune response to all investigated TAAs (37). The three selected TAAs, Survivin, Mammaglobin-A, and HER2, were chosen for their known over-expression in BC (38–40) and for their ability to evoke functional T-cell responses, measured by IFN-γ ELISpot assays, in BC patients affected by a locally-advanced tumors (24). However, the main limitation of this approach is the availability of a relatively broad panel of tumor-specific antigens selection able to reveal functional anti-tumor T-cell responses in the majority of patients (41). To obviate this limitation, we also characterized the whole TCR repertoire of circulating T-cells to detect possible changes in TCR clonality and diversity as a possible reflection of dynamic modifications in anti-tumor T-cell immunity. Intriguingly, patients showing a positive anti-TAA immune response, all presenting a low number of CTCs, displayed the highest TCR clonality, compared to cases with a level of CTCs above the threshold or low CTCs but no TAA-specific immunity. The extent of TCR clonality revealed the presence of expanded T-cell clones (42), and thus, potentially, a tumor-specific immune response. It can be hypothesized that the most expanded clones could include the TAA-specific T-cells we detected in the blood of the same patients. Unfortunately, the TCR repertoire analysis still does not allow the identification of the antigen specificity of T-cells and the immunological features of individual clones, such as the effector/exhaustion phenotype, or the CD4/CD8 expression (42, 43). Intriguingly, using single-cell RNA-sequencing, Brechbuhl et al. predicted an enhanced immune evasion in a subpopulation of BC CTCs and the enrichment of transcripts indicative for the activation of the PD‐1/PD‐L1 axis and T-cell exhaustion in T-cells isolated from patients compared with those obtained from healthy donors (44). By combining the characterization of the TCR repertoire with functional tools as the ELISpot assay, the present analysis could partially overcome this limitation, even if more complex technologies coupling single cell TCR sequencing and DNA-barcoded peptide-MHC multimer technology were recently described to fill this gap (42). In addition, the outstanding technological advances reached in the field of single-cell genomics and transcriptomics, both at the molecular and bioinformatic level, could allow the possible application of CTCs as a dynamic resource for the screening of TAA presented on CTCs or the identification of neoantigens on CTCs that might influence the efficacy of several treatments, as immunotherapies (8, 16).

On the other hand, the analysis of the TCR repertoire in the peripheral blood can provide valuable data on T-cell richness and clonality, two features that are becoming increasingly relevant as biomarkers predictive of the response to immune checkpoint inhibitors and also to chemotherapy in various cancer types including BC (45, 46). Indeed, peripheral T-cell richness/clonality can represent a surrogate measure for the ability to mount an effective anti-tumor immune response following therapy, since peripheral T-cells are a reservoir of tumor-reactive T-cells (46). Our data show that patients responding to therapy were characterized by a higher TCR clonality at baseline compared to those undergoing a progressive disease. Interestingly, all these patients had a CTC number lower than the cut-off, thus suggesting that the higher TCR clonality could reflect a more functional adaptive immunity able to counteract tumor cell metastatic dissemination (30). Notably, CTCs might be able to exploit several immune-evasion mechanisms, including: i) downregulation of MHC class I molecule (31), ii) the expression of immune checkpoints regulators, such as the programmed cell death-1 ligand 1 (PDL-1) receptor and CD47, and iii) the induction of T-cell apoptosis through the FAS/FASL pathway (47–51).

Our results also indicate that a higher clonality at baseline seemed to predict a better survival, even if the low number of patients included in the present study prevented the possibility to reach statistical significance. Other studies reported a defective anti-tumor immunity in the presence of a limited diversity in the peripheral TCR repertoire (52), concluding that a high TCR diversity may be indicative of a functional immune system with a better ability to orchestrate a broad and effective anti-tumor response (53). However, the TCR diversity can be influenced also by several biological features such as age (53) and tumor biology (54, 55). In our case study, no correlations between TCR repertoire and age was found, probably because of the limited number of patients investigated. We noted a higher TCR richness in luminal tumors compared to HER2-positive or TNBC cases, supporting the potential contribution of the biological features of these tumors in shaping the TCR repertoire (54). Conversely, the number of metastatic sites did not seem to engrave the TCR richness, while in other studies, a severe restriction of TCR diversity (divpenia) strongly correlated with the number of metastatic sites involved (55).

Several studies documented a relevant contribution of therapy to the evolution of the TCR repertoire (43, 45, 54). A clonal expansion of TILs was reported in BC patients showing a clinical response to neoadjuvant chemotherapy, thus suggesting that TIL characterization during cancer treatment may favor the identification of predictive immune biomarkers (43). A main limitation of the present study is the lack of intratumoral analysis, due to the unavailability of biopsy samples. Nevertheless, several lines of evidence demonstrated the existence of an overlapping blood-tumor TCR repertoire (42) and suggested that the circulating TCR repertoire could represent a valuable biomarker to assess the response to therapy in BC, especially for long-term immune monitoring (45). In our analysis, the number of previous treatments did not impact on the baseline TCR repertoire and the presence of a target therapy together with chemotherapy did not influence the TCR clonality at follow-up, but a global increase in expanded clones was observed after treatment. Unfortunately, due to the high variability of the therapies administered and to the limited number of patients investigated, we could not stratify patients based on the specific treatments and assess whether some drugs could have a major impact on TCR evolution. However, a higher TCR clonality was observed at follow-up in cases responding to therapy compared to progressive diseases, thus suggesting that the potential chemotherapy-induced immunomodulation can favor the expansion of an effective anti-tumor immune response (45).

Finally, the high levels of post-therapy TCR clonality that significantly correlated with therapy response, CTC numbers under the cut-off, and longer survival, supported the potential prognostic role of this promising immune biomarker.

In conclusion, the results of this exploratory study suggest a clinical relevance of the combined analysis of CTCs and anti-tumor T-cell immunity in the peripheral blood to improve the management of mBC patients, also for their eligibility to immunotherapy and their monitoring during treatment. This is particularly relevant for BC, which has showed an heterogeneous response to immune checkpoint inhibitors, with a better performance in TNBC, and thus highly requires predictive biomarkers of efficacy (56). Even if our cohort were mainly characterized by luminal subtypes, our analysis could favor the identification of patients eligible for immune checkpoint inhibitors, since recent evidence reported a potential efficacy of neoadjuvant chemotherapy and immunotherapy also in a subgroup of luminal BC (57). Application of this strategy to a larger prospective cohort of mBC patients (but also to other BC stages or different cancer backgrounds), will validate the predictive and/or prognostic relevance of this promising immune-oncological biomarker.

## Data availability statement

The original contributions presented in the study are included in the article/**Supplementary material**. Further inquiries can be directed to the corresponding author. NGS data publicly is available with the following DOI 10.21417/EM2022FO at url clients.adaptivebiotech.com/pub/muraro-2022-fo.

## Ethics statement

The study was approved by the Institutional Review Board with number IRB-12-2014. The patients/ participants provided their written informed consent to participate in this study.

## Author contributions

EM and GB conceptualized this study, carried out the experiments and analysis, drafted the initial manuscript, and revised the manuscript; FDB and RD critically reviewed the manuscript for important intellectual content; MT collected and discussed data; DC, MB, RZ, ER contributed to CTC analysis; SS recruited patients and collected clinical data; AS discussed data, supervised and contributed to conceive the study. All authors contributed to manuscript revision, read, and approved the submitted version.

## Funding

This work was supported by the Italian Ministry of Health (Ricerca Corrente) (no grant number provided) and 5x1000 per la Ricerca Sanitaria.

## Acknowledgments

We are grateful to the staff of CRO-biobank for their support in patient recruitment and sample management/preparation and to the staff of the genomic platform at CRO. We are also grateful to the physicians, the nurses of the Department of Medical Oncology at CRO and the patients who participated in this project. A special thanks goes to Prof. Alfonso Colombatti for his intellectual contribution.

## Conflict of interest

FDB, MT own shares of a start-up company with exclusive license of the patent number ITRM20130700A1, 19 Dec 2013. Patent family ID 50073355 (Published as CN105849559A; CN105849559B; EP3084434A1; EP3084434B1; ES2673597T3; WO2015092726A1; ITRM20130700A1; JP2017502312A; JP6437009B2; US2017003306A1; US9958463B2).

The remaining authors declare that the research was conducted in the absence of any commercial or financial relationships that could be construed as a potential conflict of interest.

## Publisher’s note

All claims expressed in this article are solely those of the authors and do not necessarily represent those of their affiliated organizations, or those of the publisher, the editors and the reviewers. Any product that may be evaluated in this article, or claim that may be made by its manufacturer, is not guaranteed or endorsed by the publisher.
