# Supplementary material for: Clinical relevance of the combined analysis of circulating tumor cells and anti-tumor T-cell immunity in metastatic breast cancer patients
Source: Front Oncol. 2022 Aug 23;12:983887. doi: 10.3389/fonc.2022.983887 (PMC9446887; doi:10.3389/fonc.2022.983887)
Supplement: Supplementary file 1 [file DataSheet_1.pdf]

Supplementary Material

1 Supplementary Figures and Tables

1.1 Supplementary Figures

Supplementary Figure 1.

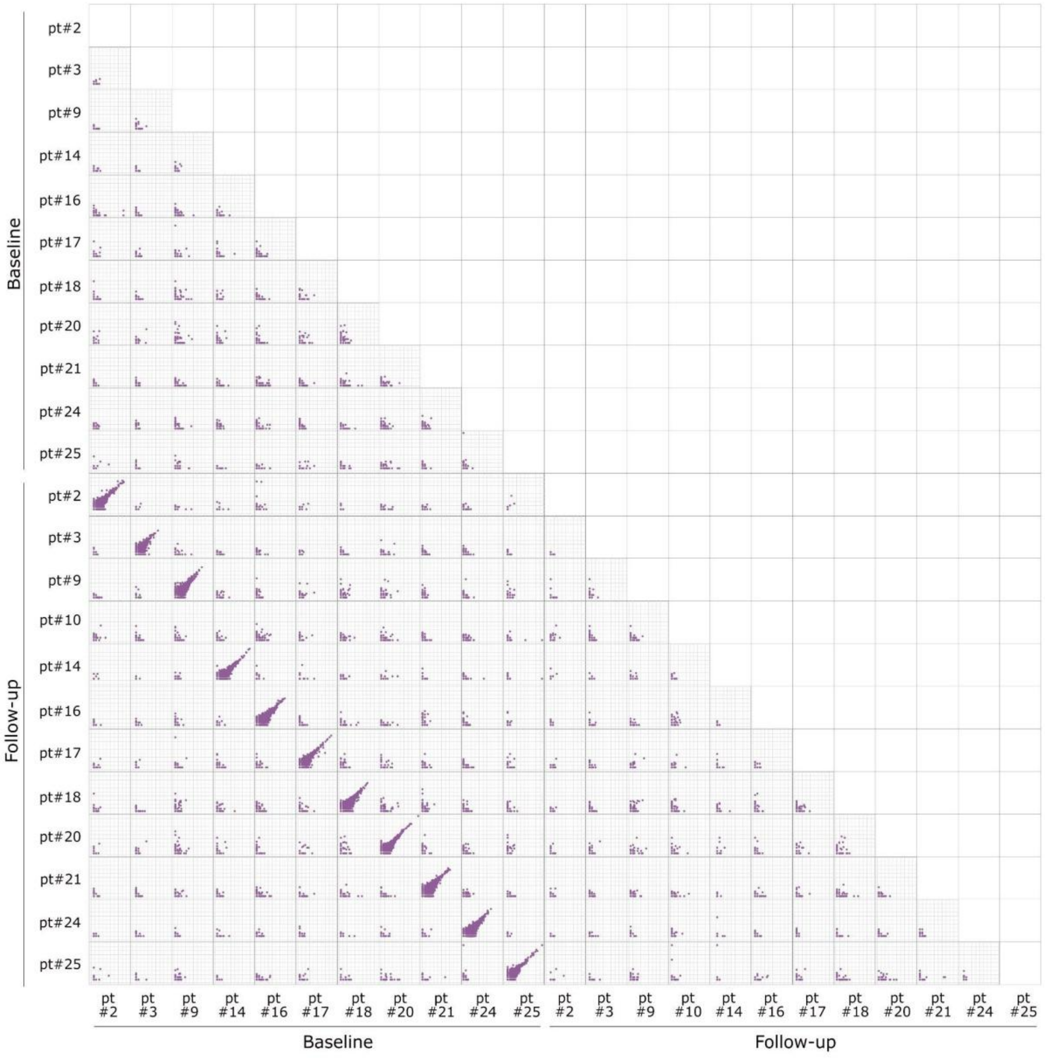

Supplementary Figure 2. Scatter plot matrix showing the extent of TCR rearrangement sharing between every sample compared with all the other samples. Each rearrangement is plotted by frequency on a log scale.

Supplementary Figure 2

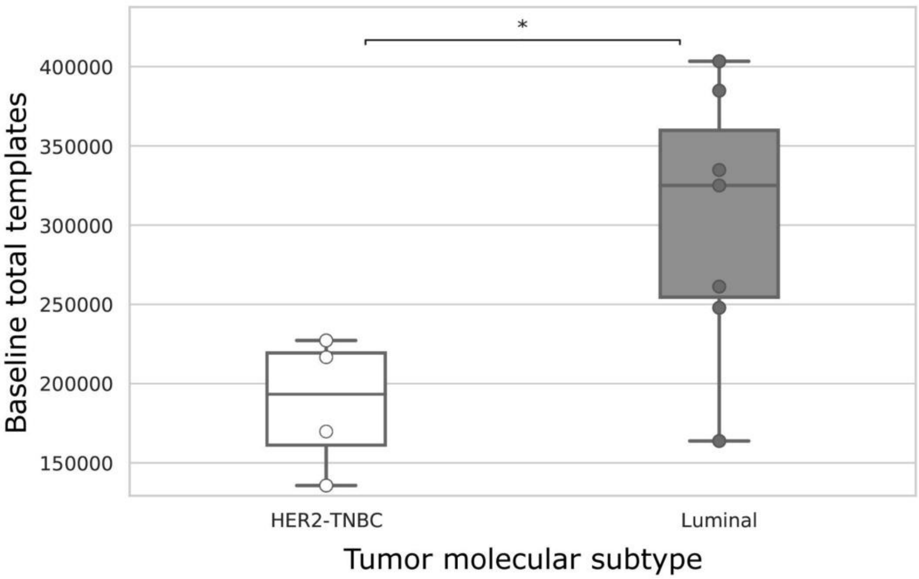

Supplementary Figure 2 Group comparison of TCR richness level between mBC patients with HER2 positive or TNBC (n=4) against luminal (n=7) tumor molecular subtype. For each boxplot, points correspond to a single sample; * p-value < 0.05.

Supplementary Figure 3

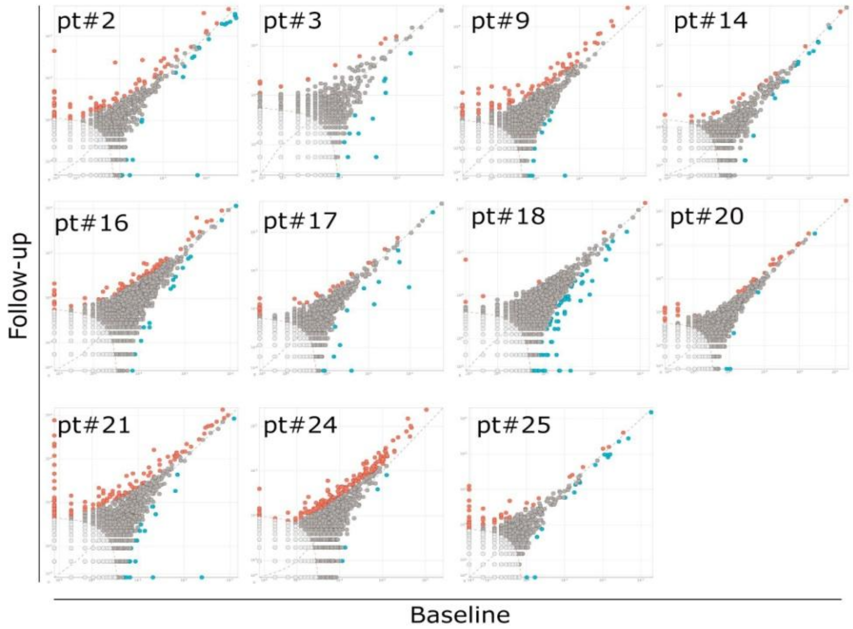

Supplementary Figure 3 Representative scatter plots showing T-cell clones differential abundance between baseline and follow-up time points of paired samples. Each unique rearrangement that has significantly increased/expanded or decreased is represented as an orange and blue point, respectively, whereas no significant changes in frequency are shown in gray.

1.2 Supplementary Tables

Supplementary Table 1. Correlative analysis based on clinical-pathological features and CTC level.

Clinical-pathological features	CTC<6	CTC ≥6	Statistical analysis
**Age**	*Median (range)*	*Median (range)*	*p-value*$^{\phi}$
	56 (43-74)	63 (40-75)	0.94
**Sex**	*n (%)*	*n (%)*	*Odds ratio (p-value)**
Female	12 (60)	7 (35)	n.a. (0.40)
Male	0 (0)	1 (5)	
**Tumor molecular subtype**	*n (%)*	*n (%)*	*Odds ratio (p-value)**
Luminal	9 (45)	4 (20)	3.0 (0.36)
HER2-pos + TNBC	3 (15)	4 (20)	
**Number of metastatic sites**	*n (%)*	*n (%)*	*Odds ratio (p-value)**
1	3 (15)	1 (5)	2.3 (0.62)
≥2	9 (45)	7 (35)	
**Type of therapy**	*n (%)*	*n (%)*	*Odds ratio (p-value)**
	10 (50)	7 (35)	0.0 (0.51)
Chemotherapy alone	2 (10)	0 (0)	
Chemotherapy and targeted therapy			
**Nr of previous treatments**	*n (%)*	*n (%)*	*Odds ratio (p-value)**
1	7 (35)	2 (10)	17.5 **(0.04)**
≥2	1 (5)	5 (25)	
**Therapy Response**	*n (%)*	*n (%)*	*Odds ratio (p-value)**
CR + PR + SD	10 (50)	1 (5)	35.0 **(<0.01)**
PD/none	2 (10)	7 (35)	

Abbreviations: CTC, circulating tumor cells; HER2, Human Epidermal Growth Factor Receptor 2; TNBC, Triple Negative Breast Cancer; CR, Complete Response; PR, Partial Response; SD, Stable Disease; PD, progressive disease; $^{\phi}$ Mann-Whitney U test; * Fisher exact test; p-values in bold are significant (p<0.05).

Supplementary Table 2. CTC enumeration, T-cell specific responses against breast tumor-associated antigens (TAA) and TCR clonality for each mBC patient analyzed before and after treatment.

ID mBC patient	MBA-CTC		TAA T-cell response at T0				TCR clonality		Tumor molecular subtype	N° of metastatic sites	Therapy response	2-years Overall survival (days)
	**T0**	**T1**	**Sur**	**Mam**	**HER2**	**Global**	**T0**	**T1**				
**1**	0	0	neg	neg	neg	neg	n.a.	n.a.	TNBC	1	CR	730
**2**	375	280	neg	neg	neg	neg	0.1112	0.0958	TNBC	1	PD	184
**3**	5319	243	neg	neg	neg	neg	0.0089	0.0082	TNBC	>2	none	43
**4**	14	0	neg	neg	neg	neg	n.a.	n.a.	TNBC	>2	PR	589
**6**	55	n.a.	neg	neg	neg	neg	n.a.	n.a.	TNBC	2	PD	208
**7**	13	4	neg	neg	neg	neg	n.a.	n.a.	Luminal	>2	PD	278
**9**	0	4	pos	neg	neg	neg	0.0176	0.0379	Luminal	>2	PR	730
**10**	0	38	pos	pos	pos	pos	n.a.	0.0262	Luminal	2	PD	234
**12**	22	5	neg	neg	neg	neg	n.a.	n.a.	Luminal	>2	PD	730
**13**	0	3	neg	neg	neg	neg	n.a.	n.a.	Luminal	2	PR	730
**14**	0	0	neg	neg	neg	neg	0.116	0.1195	TNBC	2	PR	730
**15**	0	0	neg	neg	neg	neg	n.a.	n.a.	Luminal	1	PR	312
**16**	0	133	neg	pos	neg	neg	0.0218	0.0238	Luminal	>2	PR	730
**17**	0	0	neg	neg	neg	neg	0.0766	0.0722	HER2+	1	SD	730
**18**	67	4	neg	neg	neg	neg	0.0208	0.021	Luminal	2	PD	288
**19**	0	n.a.	neg	neg	pos	pos	n.a.	n.a.	Luminal	2	PR	730
**20**	0	3	neg	pos	pos	pos	0.2052	0.2194	Luminal	>2	PR	730
**21**	0	0	neg	neg	pos	pos	0.0236	0.0295	Luminal	2	PD	217
**24**	6	4	neg	neg	neg	neg	0.0165	0.0362	Luminal	>2	PD	164
**25**	0	0	neg	pos	neg	pos	0.2003	0.1655	Luminal	>2	PR	730
N	*20*	*18*	*20*	*20*	*20*	*20*	*11*	*12*	*20*	*20*	*20*	*20*
Median	*0*	*4*	-	-	-	-	*0.024*	*0.037*	-	-	-	-
IQR	*0-16*	*0-5*	-	-	-	-	*0.02-0.11*	*0.03-0.10*	-	-	-	-
Min-Max	*0–5319*	*0–280*	-	-	-	-	*0.009-0.205*	*0.008-0.219*	-	-	-	-

Abbreviations: T0, baseline; T1, follow-up; MBA, metabolism-based assay; TAA, tumor-associated antigens; TCR, T-cell receptor; CTC, circulating tumor cell; Sur, Survivin; Mam, Mammaglobin A; TNBC, triple negative breast cancer; PD, progressive disease; PR, partial pesponse; CR, complete response; IQR, interquartile range.
